# Game-based health education to improve ART adherence of newly diagnosed young people with HIV: protocol for a stepped-wedge design randomized controlled trial

**DOI:** 10.1186/s12889-022-14708-2

**Published:** 2022-12-03

**Authors:** Min Tian, Yu Zheng, Longsheng Xie, Wei Wei, Xingli Yu, Yanhua Chen, Jian Tang

**Affiliations:** 1 Department of Orthopedics, The Affiliated Hospital of Southwest Medical University, 25 Taiping Street, Luzhou, China; 2 Department of Rheumatology and Immunology, The Affiliated Hospital of Southwest Medical University, 25 Taiping Street, Luzhou, China; 3 Department of Nephrology, Affiliated Hospital of Southwest Medical University, 25 Taiping Street, Luzhou, China; 4 School of Nursing, Southwest Medical University, 1 Xianglin Road, Luzhou, China; 5 Department of Operating Room, The Affiliated Hospital of Southwest Medical University, 25 Taiping Street, Luzhou, China; 6 Department of Nursing, The Affiliated Hospital of Southwest Medical University, 25 Taiping Street, Luzhou, China

**Keywords:** Game-based education, Young people infected with HIV, Antiretroviral therapy, Adherence

## Abstract

**Background:**

Antiretroviral therapy (ART) is one of the most effective ways for HIV-infected to treat AIDS. However, it is difficult to start ART among young people those newly diagnosed HIV-infection in China, and their adherence to ART is poor. We have designed an AIDS educational game called AIDS Fighter · Health Defense, which could improve the AIDS-related knowledge and has the potential to improve AIDS prevention ability of young students. In this study, AIDS Fighter · Health Defense will be used with newly diagnosed young people with HIV to evaluate the education effect of the game in improving ART adherence.

**Design:**

A stepped-wedge design randomized controlled trial will be conducted to confirm the education effect of AIDS Fighter · Health Defense on improving ART adherence of newly diagnosed young people with HIV, and to verify when to start game-based health education could be more effective for newly diagnosed young people with HIV.

**Methods:**

Participants will receive AIDS education from health workers and start ART when diagnosed with HIV and assigned into four groups randomly. The first step group to the fourth step group will receive AIDS Fighter · Health Defense in turn at the star of ART, one week, one month and three months after the start of ART. The primary outcomes are medication adherence, CD4( +) T cell count, and HIV viral load. The secondary outcomes are ART-related knowledge, ART-related skills, psychological resilience, and self-discrimination. Assessments will be completed before the intervention and one week, first month, and third month of the intervention, and then a one-year follow-up evaluation will be conducted after the intervention.

**Discussion:**

AIDS Fighter · Health Defense may be an effective approach to help newly diagnosed young people with HIV to improve ART adherence. A stepped-wedge design randomized controlled trial of this study may find the optimal time of AIDS education to improve ART adherence of newly diagnosed young people with HIV.

**Registration number:**

Chinese Clinical Trial Registry: ChiCTR2200059766, registered 11 May 2022. http://www.chictr.org.cn/showproj.aspx?proj=169420

**Supplementary information:**

The online version contains supplementary material available at 10.1186/s12889-022-14708-2.

## Background

Antiretroviral therapy (ART) is one of the most effective treatments for HIV-infected [1]. There is a global consensus that adherence to ART is critical to the success of HIV treatment [2, 3]. Good ART adherence of HIV-infected could help them maintain viral suppression [4], reduce HIV contagiousness [5], maintain high CD4( +) T cell count and reduce the incidence of AIDS-related complications [6]. In China, 92.6% of people live with HIV (PLHIV) had access to ART by the end of 2021 [7]; And a study of 1198 PLWH from Guangxi, China, showed 80.3% of people receiving ART had optimal medication adherence [8]. While some progress has been made in ending the AIDS epidemic, another point of concern should be noted, namely the persistence of ART adherence [9]. A meta-analysis of ART adherence in China showed that, the mean ART adherence among HIV-infected in China was 81.1% at one week of ART initiation, 80.9% at one month of ART initiation, and dropped to 68.3% at three months of ART initiation [10]. Which means that a sharp decline occurred within three months, making it more difficult to adhere to long-term antiviral treatment.

Adolescents have lower adherence rates than children and adults [11–13]. According to a Ugandan study, only 63.4% of young people are adhered to their ART consistently [14]. In 2021, there are over 1.14 million cases of HIV infection, of which young people aged 15–25 account for about 36% [15, 16]. Even among those newly infected, approximately 28% are young people [17]. Moreover, it is difficult for newly diagnosed young people with HIV to start ART. A survey showed that only 39.5% of young people infected with HIV could initiate ART in a timely manner [18], and ART adherence was poor after initiation [19]. Lack of ART related knowledge is one of the important reasons affecting the medication behavior of young people [20]. AIDS health education has been shown to be an effective way to improve the self-management ability of young people infected with HIV [21]. However, there is a lack of health education on ART adherence among newly diagnosed young people with HIV [22].

Game-based health education has been widely used in disease education, like diabetes [23], breast cancer [24], cardiovascular disease [25], etc., and has been shown to have good educational effects. Game-based health education on AIDS was mainly aimed at young people [26], including HIV-infected adolescents, gay men, and other high-risk groups [27, 28]. The goals of education were to increase the adherence with pre-exposure prophylaxis (PrEP) and ART [29, 30], promote AIDS screening [31], reduce risky sexual behaviors and avoid drug and alcohol abuse [32–34]. However, there were few studies on game-based health education on ART in newly diagnosed young people with HIV.

We have developed an AIDS educational game called AIDS Fighter· Health Defense, which has seven levels with difficulty increasing as the levels continue. The storyline of the game is that HIV has invaded the human body, and players need to fight to clear HIV. Players are required to collect ART medicine and condoms, avoid HIV, alcohol and drug, take ART medicine regularly, and answer questions about ART. More information about the game can be found in our published studies [35, 36]. It had been shown that the game can improve the AIDS-related knowledge and has the potential to improve AIDS prevention ability of young students. Therefore, we believe that AIDS Fighter· Health Defense could be an effective, large-scale intervention tool for AIDS health education. In this study, AIDS Fighter · Health Defense will be used with newly diagnosed young people with HIV to evaluate the education effect of the game in improving ART adherence.

Below we describe the protocol of our stepped-wedge design randomized controlled trial to evaluate the effect of the game to improve the ART adherence of newly diagnosed young people with HIV. The trial is a 3-month game-based intervention, and the game includes components designed to increase ART adherence, ART knowledge, ART skills, psychological resilience and perceived benefits, and to reduce self-discrimination, anxiety and depression.

## Methods/Design

### Study design

A stepped-wedge design randomized controlled trial with four groups will be conducted. Which is a relatively new research design [37]. The design included an initial phase in which no groups are exposed to the intervention. Subsequently, at fixed time intervals (“steps”), a group (or set of groups) is randomly crossed over from the control to the intervention under evaluation. The start of the intervention is different for each group, and this design helps to verify the effect of intervention timing on intervention effect.

**Fig. 1:**
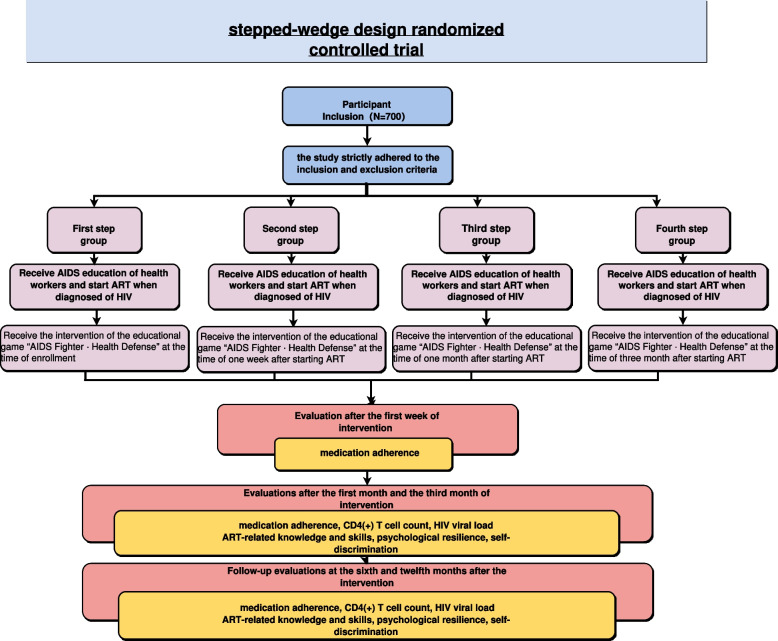
Study workflow diagram

Participants will be assigned to the four groups in 1:1:1:1 randomly. There is no dedicated control group in this study, and each step group will receive AIDS Fighter · Health Defense intervention in turn as the trial progress (see Fig. 1 for study workflow diagram). The group which has not received the intervention will be treated as the control group. Data collection will be conducted before and after intervention in each group. Participants in each group will be evaluated for medication adherence after the first week of intervention and the other outcomes will be evaluated after the first month and third month of intervention. Follow-up evaluations for the same outcomes will be performed at 6 and 12 months after the end of the intervention.

Our hypotheses are:

1. Compared with the control groups, those in the AIDS Fighter · Health Defense intervention groups will have better ART adherence.
2. Varying time to receive AIDS Fighter · Health Defense intervention may lead to varying ART adherence.
3. AIDS Fighter · Health Defense could help newly diagnosed young people to maintain good long-term ART adherence.

### Intervention design

**Fig. 2:**
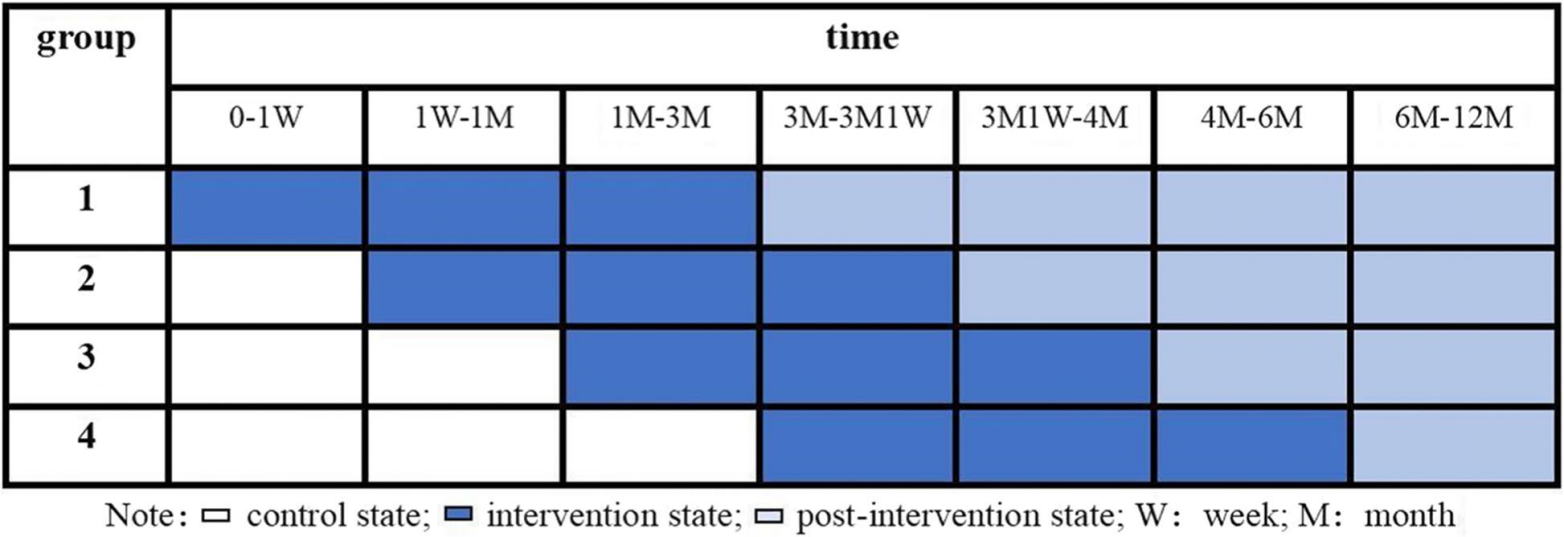
Intervention design

Participants will receive AIDS education from health workers and start ART when diagnosed of HIV and randomly assigned into four groups. The first step group to the fourth step group will receive AIDS Fighter. Health Defense in turn at the start of ART, one week after the star of ART, one month after the star of ART and three months after the star of ART. Assessments will be completed before the intervention and after the first week, first month, and third month of intervention, and then a one-year follow-up evaluation will be conducted. The intervention design is shown in Fig. 2.

### The first step intervention group

The first step group will receive the intervention of the educational game “AIDS Fighter · Health Defense” through WeChat at the time of enrollment and will be required to play the game at least 5 days a week and 20 min a day. During the 3-month intervention, participants will receive daily message notifications that inform users to play the game. A data management system will be used to monitor participants' time in the game and check details of the participant's use of the game.

### The 2-4 step intervention group

The intervention of 2-4 step groups will be same as the first step intervention group, but the time they receive AIDS Fighter · Health Defense will be different. The second step group will receive the intervention 1 week after starting ART. The third step group will receive the intervention 1 month after starting ART. The fourth step group will receive the intervention 3 months after starting ART.

### Recruitment of participants

The participants in this study are newly diagnosed young people with HIV. On-site recruitment will be conducted in AIDS clinics in Sichuan Province, China, where there are more than 10000 newly diagnosed people a year [38]. The inclusion criteria are: 1) diagnosed with HIV within 30 days [18]; 2) Young people aged 15 to 24; 3) Participants have clear consciousness and are able to read and communicate; 4) Participants were informed and voluntarily participated in the study; 5) Parental consent will be required to use the game if the participants are under 18 years old. And adults need to declare that they can use the game when they sign the informed consent. Those who have played similar games before will be excluded from the study. When the participants meet the inclusion criteria and agree to join this research, informed consent form will be signed.

### Sample size calculation

The sample size was performed using the formula developed by Hemming [39]. The basic parameters for sample size calculation are based on the mean rate of adherence to ART at one week (81.1%) [9], assuming that the ART adherence of the intervention group would reaches 95%. The significance level of the test is α=0.05, the test power is 1-β=0.9, the coefficient of variation is ρ=0.15, the number of observation periods is t+1=7, the number of steps is t=6, the number of clusters is k=4, and N1 is calculated based on the sample size of the two groups. PASS software was used to calculate the initial sample size N1=220. According to the following formula:

$$\begin{array}{c}a=2k\left(t-\frac{1}{t}\right)\rho \left(1+\frac{t}{2}\right)\\ b={3N}_{1}\left(1-\rho \right)\rho \left(1+t\right)-2k\left(t-\frac{1}{t}\right)\left(1-\rho \right)\\ \begin{array}{c}c={3N}_{1}{\left(1-\rho \right)}^{2}\\ m=\frac{-b\pm \sqrt{{b}^{2}-4ac}}{2a}\\ N=\mathrm{m}\times \left(t+1\right)\times k\end{array}\end{array}$$

The required sample size for each group is calculated to be 140. Considering a potential 20% loss to follow-up, the final sample size for each group is 175, and the total sample size is 700.

### Randomization and allocation concealment

Opaque envelopes will be used for randomization. Among the 700 envelopes, each envelope contained a note with the words "First Step Group", "Second Step Group", "Third Step Group" and "Fourth Step Group" (175 envelopes per group). An opaque envelope will be selected for the first time the participant enrolled, and they will be assigned to the corresponding group according to the envelope. The health workers for the AIDS education of newly diagnosed young people with HIV and the data analysts will be blinded for the allocation.

### Quality control

Before the start of the trial, a non-disclosure agreement needs to be signed of all participants. They will be required to keep the contents of the intervention strictly confidential to ensure each group will receive the game at the specified time. Pre-testing will be conducted before the study begins, and the formal study protocol will be adjusted based on the results of the preliminary tests. After completing the research, each participant will receive a cash prize ranging from 20 to 100 RMB through a lottery.

### Outcomes and data collection

The primary outcomes are medication adherence, CD4( +) T cell count, and HIV viral load. Medication adherence will be assessed by calculating the Medication Possession Ratio (MPR) [40], which will be calculated by the following formula:

$$MPR=\frac{days\mathrm{cov}eredwiththesumofdailydoses}{daysonART}$$

MPR is defined from 0 to 1, with closer to 1, indicating higher the adherence. In addition, The CPCRA 7-day recall adherence questionnaire [41] will be used to assess the medication adherence. Within the questionnaire, participants will be asked to recall the proportion of the prescribed medication in the past 7 days (100%, 80%, 50%, 20%, 0%) and the amount of missed medication in the past 3 days, 7 days and 30 days. CD4( +) T cell count, HIV viral load will be obtained by biochemical testing, and the testing will be carried out in a medical unit with testing qualifications. Participants with less than 400 copies/mL will be coded as being virally suppressed, while participants with viral loads of 400 or more copies/mL will be classified as not being virally suppressed [42].

Secondary outcomes are ART-related knowledge, ART-related skills, psychological resilience, self-discrimination. ART-related knowledge will be assessed by ART-related knowledge questionnaire [ 43], which was developed in Chinese with 21 items, each item has a correct answer and scored one point. A higher the score means higher the ART-related knowledge. ART-related skills will be assessed through a self-made questionnaire with 20 items, one point being allocated for a correct answer. The higher the score, means higher the ART-related skills. Psychological resilience will be assessed by the Resilience Scale for PLHIV [44], which was developed by our study team, including three dimensions and 29 items, one point for a correct answer. A higher the score means higher psychological resilience. Self-discrimination will be assessed by the Chinese version of Berger HIV stigma scale [45], which has 18 items, each item scored on a 4-point scale (from "strongly disagree" to "strongly agree" with a score of 1 to 4). A higher the score means higher self-discrimination.

### Data analysis

All data will be uniformly entered by Epidata 3.1. And SPSS 26.0 statistical software will be used for analysis. The categorical data will be described by composition ratio and compared by chi-square test; quantitative data will be described by mean ± standard deviation and compared by Analysis of Variance; Generalized estimating equations models and linear mixed-effect (LME) models will be used to analyze the effect of game-based health education on ART medication adherence, CD4( +) T cell count, ART-related knowledge, psychological resilience, and self-discrimination. Mixed effects logistic regression models will be used for HIV viral load outcomes. Difference-in-difference analysis will be used to evaluate the change in the intervention groups excluding the effect of the changing over time. The differences are statistically significant with P ≤ 0.05.

### Ethical approval and clinical trial registration

This study has been approved by the Ethics Committee of The Affiliated Hospital of Southwest Medical University, with the approval number KY2022163. And has been registered at Chinese Clinical Trial with the registration number ChiCTR2200059766. The Ethics Committee will audit the conduct of the trial, and have the right to make the decision to terminate the trial. The process will be independent from investigators and the sponsor. The study data will be uploaded to the China Clinical Trials Registry after the study is complete.

## Discussion

The adherence of ART is fine in developed countries or areas, but which is poor in the southwest of China [46]. Many HIV-infected could not initiate ART immediately, with where the economy is relatively backward, people are poorly educated, and AIDS-related knowledge is lacking [47]. Especially for young people newly diagnosed with HIV in those areas, it is hard for them to start ART or adhere ART for long term [18, 19]. As many young people at the time they diagnosed with HIV, the immune damage was not severe and there were no clinical symptoms [48]. In addition, young people's social needs lead to their fear of being found to be taking ART medicine [49]. Therefore, it is needed to carry out HIV and ART-related health education among newly diagnosed HIV-infected people to improve their adherence of ART.

With the development of science and technology, AIDS health education has changed from traditional to modern ways. Among them, game-based intervention has become a significant method to change healthy behaviors [50, 51]. In our previous studies, an AIDS educational game called AIDS Fighter· Health Defense was developed, and a randomized controlled trial was conducted and proved that this game has a good education effect on AIDS prevention for young students. Students have good comments on the game and believe it could help them learn more about AIDS [36]. In this study, we would like to evaluate the effect of this game to improve ART adherence of young people newly diagnosed with HIV. And provide evidence for game-based education to improve ART adherence in China.

Someone diagnosed with HIV may be shocked and frustrated, especially young people who need to live with HIV for a long time [52]. It will take a while for them to adjust to the fact of being infected with HIV and accept to start ART [53], but they usually do not get that time and receive AIDS education and are required to start ART at the time of diagnosis in China [54]. This might be one of the reasons why adherence to ART sharply declines in the short term after initiation [55]. ART-related education could be more effective in maintaining long-term ART adherence in newly diagnosed people with HIV after they accept the fact of infection and are willing to learn AIDS-related knowledge [56, 57]. But it is limited to know how long it will take for young people infected with HIV to recover psychologically after infection. Therefore, we designed the protocol to find the optimal time point of intervention for ART adherence education. In this study, game-based education will be added to regular AIDS education to improve ART adherence of newly diagnosed young people with HIV. A stepped-wedge design randomized controlled trial will be conducted to evaluate the effect of game-based education and figure out when to start game-based education could be more effective. This could provide a reference for whether HIV-infected people need to be given some time for self-adjustment before receiving AIDS education and find out the difference in educational effect between different self-adjustment times.

We believe with the development of this study we will discover whether game-based education is effective in improving ART adherence in newly diagnosed young people with HIV. We could develop an AIDS education gamification method for China and other countries to improve the ART adherence of newly diagnosed young people with HIV. Beyond that, we will discover whether starting AIDS education at different times makes a difference and ascertain when to start AIDS education more effectively in China. This could be an innovation in AIDS education.

## Supplementary information

**Additional file 1.**

## Data Availability

The data collected in this study will be published in papers after the study is completed.

## Abbreviations

AIDS: Acquired Immune Deficiency Syndrome
ART: Antiretroviral therapy
CPCRA: Community Programs for Clinical Research on AIDS
HIV: Human Immunodeficiency Virus
MPR: Medication Possession Ratio
LME: Linear mixed-effect
PLHIV: People Living with HIV
PrEP: Pre-exposure prophylaxis
WHO: World Health Organization
